# Untargeted Large Volume Hyperthermia Reduces Tumor Drug Uptake From Thermosensitive Liposomes

**DOI:** 10.1109/OJEMB.2021.3078843

**Published:** 2021-05-11

**Authors:** KRISHNA K. RAMAJAYAM, A. MARISSA WOLFE, ANJAN MOTAMARRY, GEORGES J. NAHHAS, JOHN YOST, MICHAEL J. YOST, DIETER HAEMMERICH

**Affiliations:** 1 Department of Pediatrics, Medical University of South Carolina, Charleston, SC 29425 USA; 2 Ralph H. Johnson VA Medical Center, Charleston, SC 29401 USA; 3 Hollings Cancer Center, Medical University of South Carolina, Charleston, SC 29425 USA; 4 Department of Psychiatry and Behavioral Sciences, Medical University of South Carolina, Charleston, SC 29425 USA; 5 Department of Surgery, Medical University of South Carolina, Charleston, SC 29425 USA

**Keywords:** Computer models, drug delivery, hyperthermia (HT), *in vivo*, thermosensitive liposomes

## Abstract

**Goal:**

The impact of hyperthermia (HT) method on tumor drug uptake with thermosensitive liposomes (TSL) is not well understood.

**Methods:**

We created realistic three-dimensional (3-D) computer models that simulate TSL-encapsulated doxorubicin (TSL-DOX) delivery in mouse tumors with three HT methods (thermistor probe (T), laser (L) and water bath (WB), at 15 min and 60 min HT duration), with corroborating *in vivo* studies.

**Results:**

Average computer model-predicted tumor drug concentrations (*μ*g/g) were 8.8(T, 15 min), 21.0(T, 60 min), 14.1(L, 15 min), 25.2(L, 60 min), 9.4(WB, 15 min), and 8.7(WB, 60 min). Tumor fluorescence was increased by 2.6 × (T) and 1.6 × (L) when HT duration was extended from 15 to 60 min (p *<* 0.05), with no increase for WB HT. Pharmacokinetic analysis confirmed that water bath HT causes rapid depletion of encapsulated TSL-DOX in systemic circulation due to the large heated tissue volume.

**Conclusions:**

Untargeted large volume HT causes poor tumor drug uptake from TSL.

## I. INTRODUCTION

Chemotherapy is limited by systemic toxicities and inadequate tumor uptake, in part due to the typically short plasma half-life and rapid distribution of chemotherapy agents [2]. Various nanoparticle delivery systems have been developed to address these limitations, usually depending on enhanced permeability and retention (EPR) for preferential tumor accumulation of nanoparticles [2]–[4]. There is increasing evidence that nanoparticles based on EPR have reached an upper delivery limit [2], and that new strategies are required to further enhance tumor drug uptake [3].

One promising strategy are stimuli-responsive nanoparticles, where drug release is triggered by internal or external stimuli [5], [6]. Possibly the most widely researched nanoparticle falling under this category are thermosensitive liposomes (TSL), where drug release is triggered by hyperthermia (*>*40 °C) [7], [8]. Combined with image-guided hyperthermia (HT) technologies, TSL enable drug delivery to tissue regions identified by imaging [8], [9], [10].

While the first TSL formulation was described more than 40 years ago [11], most current TSL formulations are based on the more recently established intravascular triggered release paradigm, where drug is released in the microvasculature of the heated tumor [12]–[14]. This paradigm enables ~10–30 times higher tumor drug uptake compared to unencapsulated drug [8], [15], while also enhancing drug penetration [14].

Various HT technologies have been used in combination with TSL. In human patients, microwave HT and high-intensity focused ultrasound (HIFU) have been employed [9], [10], [16], [17]. In preclinical studies, additional HT methods such as laser HT, thermistor probes and water bath heating have been used [1], [8], [18], [19], with the latter the most widely used method - likely due to simplicity and availability. Very few studies directly compared the impact of HT methods on drug delivery with TSL. One study demonstrated differences in tumor drug uptake comparing three HT methods, but the reasons for these uptake differences were not identified [18]. The focus of the current study was to quantify the impact of HT methods with varying heat penetration on drug delivery with TSL-DOX.

**FIGURE 1.**
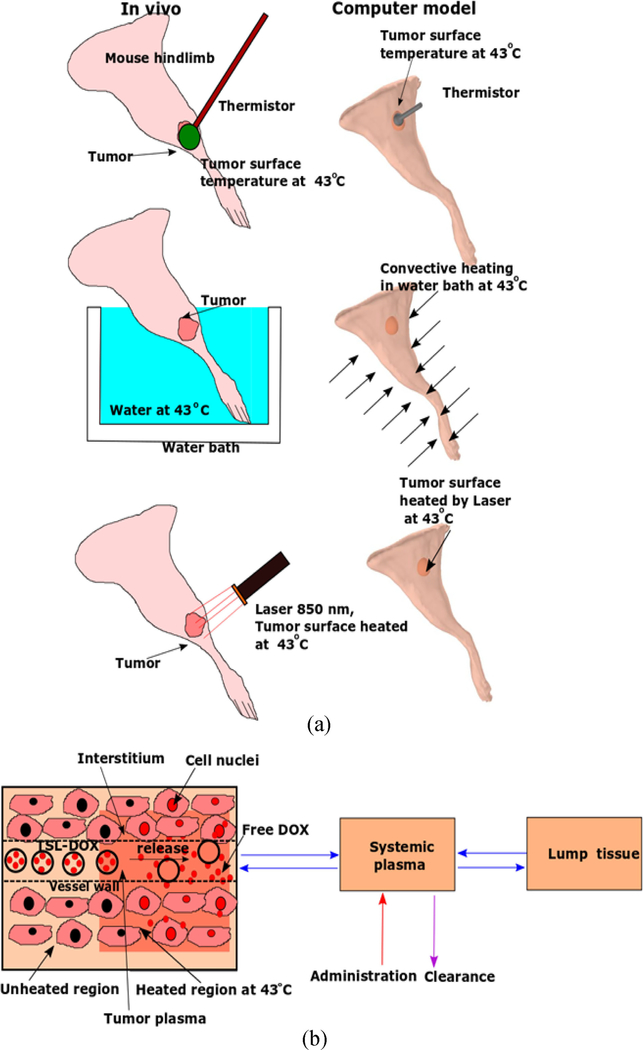
(a) Schematic representation of HT methods used *in vivo* in comparison to the computational model. (b) Overview of drug delivery computer model. TSL-DOX is administered as bolus into systemic circulation, and HT is applied. TSL-DOX enters the heated tumor region, resulting in intravascular drug release. The released (free) DOX (red dots) then extravasates into the interstitium (EES), is taken up by cells, and finally interacts with the cell nucleus (indicated as red colored cell nuclei).

Computer models enable the visualization of the 3-D distribution of both temperature and drug concentration in different tumor compartments, including how these distributions vary over time [20]–[22]. Thus, computer models provide additional information not accessible in experimental studies. In the present study, we present 3-D computational models based on the anatomically accurate geometry of a preclinical tumor model and simulated three HT methods to quantify impact on tumor drug uptake (Fig. 1). The computer model results were corroborated by *in vivo* studies where tumor drug uptake was monitored by fluorescence imaging. Based on the results, we then identify the reasons behind the observed differences in tumor drug uptake resulting from the different HT methods.

## II. RESULTS

### A. EVALUATION OF TEMPERATURE AND DRUG DELIVERY BY COMPUTER MODELS

**FIGURE 2.**
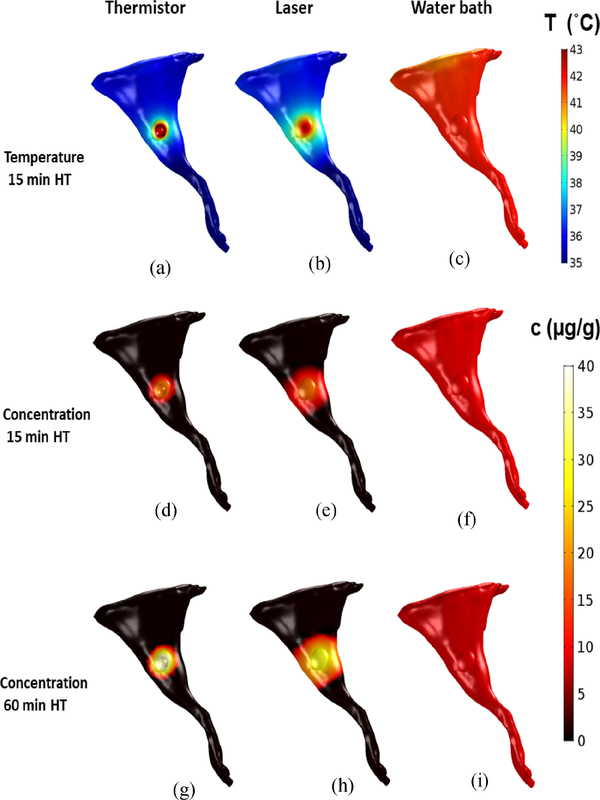
Tissue surface temperature after 15 min HT (HT) is shown for (a) thermistor, (b) laser, and (c) water bath. The resulting surface drug concentration for 15 min and 60 min HT is shown for (d,g) thermistor, (e,h) laser, and (f,i) water bath. Drug concentration is shown 10 min after completion of HT in (d-i).

The comparison of surface temperature in the 3-D model demonstrates the differences between the three heating methods. For all three methods (Fig 1(a)), temperature within the targeted tissue region of the hind limb rose rapidly after heating was initiated. Temperature reached a steady-state after 8 min (thermistor, laser) and 3 min (water bath). Both thermistor probe and laser achieved very focused heating, while the water bath provided uniform temperature throughout the limb surface (Fig. 2(a)–(c)). The computer model for drug delivery was based on a multi-compartment model (as shown in Fig 1(b), with detailed description in Suppl. Materials). This model considered experimental data on TSL-DOX release with significant TSL release occurring above ~40 °C (see Fig. S2). Accordingly, the computer model predicted focused drug delivery in the tumor region for thermistor probe and laser, and uniform drug delivery for water bath (Fig. 2(d)–(i)). Note that all plots visualizing drug uptake only consider unencapsulated drug in cellular, interstitial, and plasma compartments based on the compartmental volume fractions, and do not consider non-bioavailable, encapsulated drug. The highest drug concentrations at the tumor surface were 18.2 *μ*g/g (thermistor 15 min HT), 17.5 *μ*g/g (laser 15 min HT), and 9.5 *μ*g/g (water bath 15 min HT). Thus, while the water bath achieved the most uniform drug delivery throughout the hind limb, the concentration in the tumor region was considerably lower.

### B. COMPUTER MODEL VISUALIZES TUMOR DRUG PENETRATION

**FIGURE 3.**
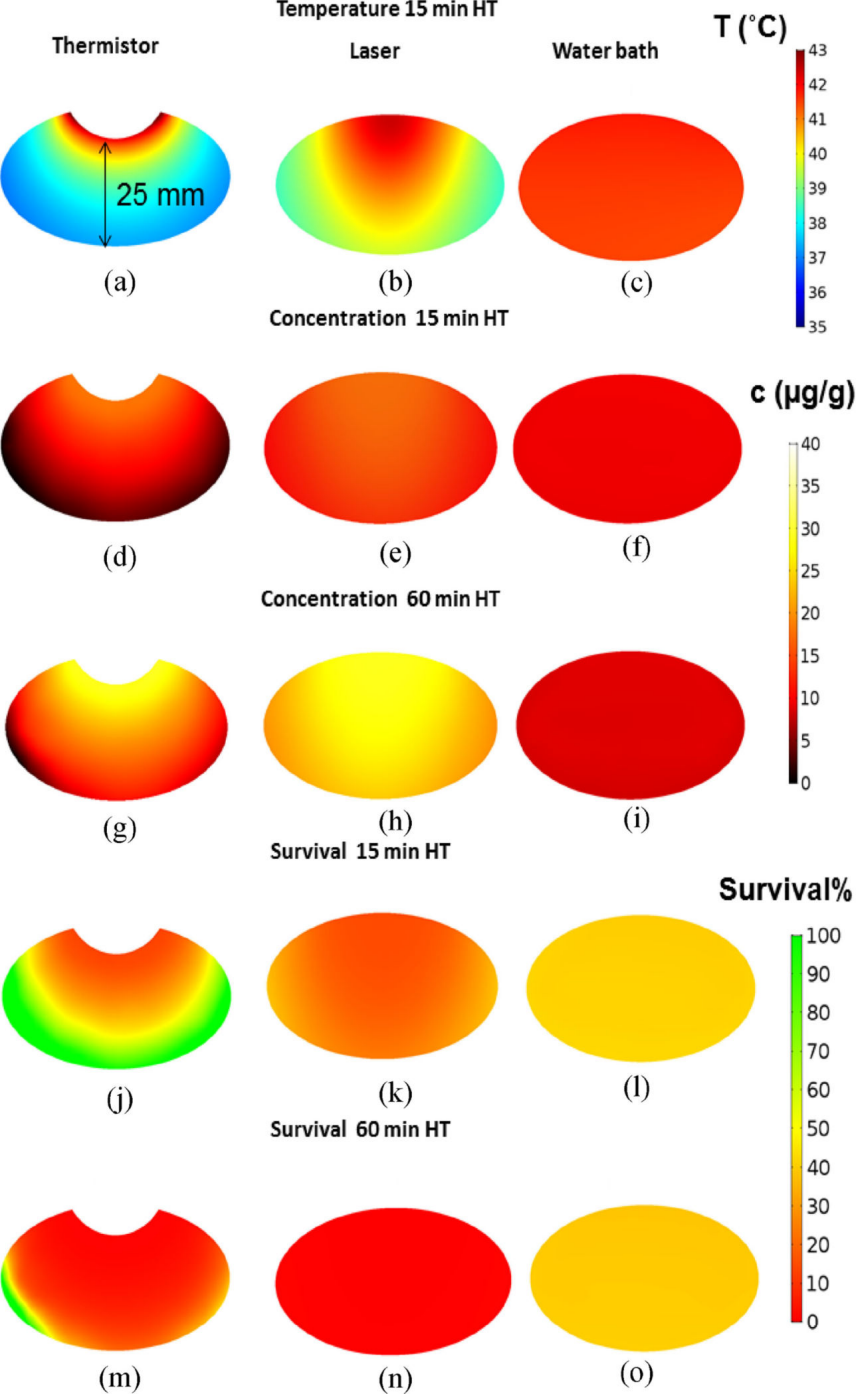
Temperature, drug concentration, and survival fraction maps in a central tumor cross section orthogonal to the leg surface (heat was applied from the tumor surface, located at the top). Tumor temperature after 15 min HT is shown for (a) thermistor, (b) laser, and (c) water bath. Drug concentration for 15 min and 60 min HT is shown for (d,g) thermistor, (e,h) laser, and (f,i) water bath heating. Tumor cell survival fraction (%) is shown after 15 and 60 min HT for (j,m) thermistor, (k,n) laser and (l,o) water bath.

Since the computer model simulated drug delivery kinetics in 3-D, we could analyze drug uptake variation across the tumor. To examine tumor drug penetration relative to the tumor surface from which heat was applied, we analyzed drug distribution centrally through the tumor, in a plane orthogonal to the leg surface. We report the tumor drug concentration maps at two time points: (1) 10 min after the end of 15 min HT and, (2) 10 min after the end of 60 min HT treatment. The thermistor probe achieved DOX concentrations near the probe of 18.5 *μ*g/g for 15 min HT (Fig. 3d)), and 36.8 *μ*g/g for 60 min HT (Fig. 3(g)). Similarly, for the laser, the achieved concentrations were 17.5 *μ*g/g for 15 min HT (Fig. 3(e)) and increased to 28.9 *μ*g/g after 60 min HT (Fig. 3(h)). Drug penetration was however significantly improved with laser compared to thermistor probe owing to better heat penetration throughout the tumor volume (Fig. 3(a),(b)). While the thermistor only achieved limited drug delivery to deeper tumor regions, the laser produced good drug uptake throughout the tumor since the infrared laser light penetrates the tissue a few mm, while the thermistor only directly heats the tumor surface. The water bath produced very uniform, but significantly lower tumor drug concentrations than thermistor or laser. DOX concentrations for water bath HT were 9.6 *μ*g/g after 15 min HT (Fig. 3(f)) and decreased to 8.9 *μ*g/g after 60 min HT (Fig. (i)), rather than increasing as observed in the other two HT methods. DOX concentrations 10 min after the cessation of HT treatment were somewhat lower than the concentration achieved at the end of heating in all cases, in part due to back-diffusion into plasma of DOX present in the interstitium, and in part due to effusion of cellular unbound drug. Average tumor drug concentrations are summarized in Table I. We also estimated tumor cell survival fraction based on data from a prior *in vitro* study [23]. Only for 60 min laser HT a potentially adequate survival fraction is predicted throughout the tumor (Table I), with likely inadequate therapeutic efficacy for the other HT conditions Fig. 3(j)–(o).

### C. COMPUTER MODEL EXPLAINS DRUG TRANSPORT KINETICS AT THE CELLULAR SCALE

**FIGURE 4.**
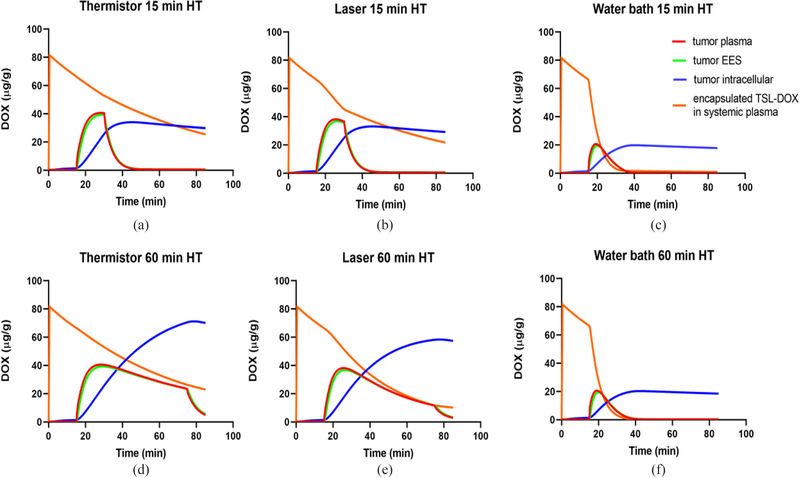
Drug concentration in individual tumor plasma, tumor interstitium (EES), and tumor cellular compartments. Concentration time course is shown for a location centered on the tumor surface, for both 15 and 60 min HT, (a,d) for thermistor, (b,e) for laser, and (c,f) for water bath heating.

Since plasma, interstitial (extra-cellular extravascular space (EES)), and cellular tissue compartments were represented in the computational model, we could follow drug transport at the cellular scale. We report the drug concentration time course in these compartments at a location centrally at the tumor surface. For all three heating methods, overall similar transport kinetics were observed: once heating starts, tumor plasma concentration of free DOX rises rapidly due to release from TSL and reaches a plateau a few minutes later. The interstitial (EES) concentration closely follows tumor plasma concentration of free drug, indicating transport of released drug from tumor plasma to EES. The DOX available in the EES is then continuously taken up by cells. Once heating stops, tumor interstitial and plasma concentrations drop rapidly, indicating cessation of TSL release and transport of drug from EES back to tumor plasma. For both thermistor and laser, extending the HT duration from 15 to 60 min produced a corresponding extension of the plateau region of tumor plasma and interstitial concentrations (Fig. 4(d), (e)). As drug concentration is now kept elevated in the interstitium and available for cellular uptake for a longer duration, total intracellular drug uptake was higher when the HT duration was extended. This was however not true for water bath heating and can be explained by the following: the systemic TSL-DOX concentration represents drug available for release and declines slowly due to leakage and elimination even without heating. In addition, drug release from TSL due to heating reduces the systemically available encapsulated drug. Importantly, this amount of drug released from TSL (and by which systemically available TSL-DOX is reduced) depends on the heated tissue volume. For thermistor and laser heating, only a small tissue volume in the tumor region is heated, resulting in a small to moderate decrease in systemic TSL-DOX (Fig. 4(a), (b)). In contrast to thermistor and laser, water bath HT is applied to the whole limb. This untargeted HT exposure to a large tissue volume rapidly depletes all systemically available TSL-DOX (Fig. 4(c)). In fact, almost all TSL-encapsulated drug is depleted after 15 min water bath HT, explaining why there is no increase in tumor drug uptake observed when extending the heating duration to 60 min (Fig. 4(f)). In addition, this rapid depletion results in a considerably smaller tumor plasma peak of 20 *μ*g/g, compared to ~35–40 *μ*g/g for laser and thermistor, explaining the significantly lower cellular uptake compared to thermistor and laser heating even for 15 min HT. The peak intracellular DOX concentration is of relevance, since it is predictive of cell survival (Fig. S1) [23], and was 26.9 *μ*g/g and 70.5 *μ*g/g (thermistor, 15 and 60 min), 24.7 *μ*g/g and 58.1 *μ*g/g (laser, 15 and 60 min), and 16.4 *μ*g/g and 18.9 *μ*g/g (water bath, 15 and 60 min).

### D. IN VIVO IMAGING VISUALIZES DRUG DELIVERY WITH DIFFERENT HT METHODS

**FIGURE 5.**
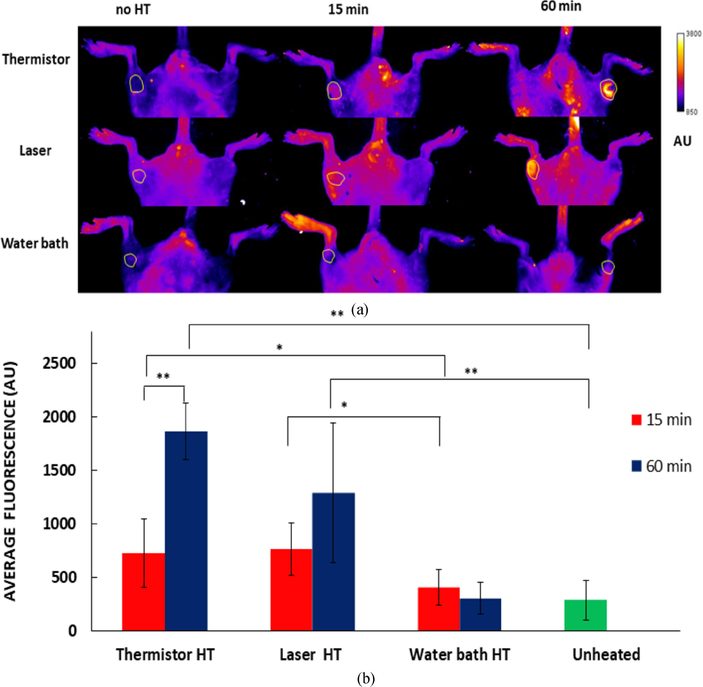
(a) In vivo fluorescence images before HT, and after HT with different heating methods (Thermistor, Laser and water bath; 15 and 60 min HT). Tumor ROI is indicated by round outline in each image. (b) Average fluorescence in the tumor ROI after HT for each treatment group, with background fluorescence (before drug injection) subtracted. Tables with fluorescence values and statistical analysis results available in Suppl. Mat. Error bars indicate standard deviation, and significant difference between groups is indicated: ∗(p *<* 0.05); ∗∗(p *<* 0.001).

*In vivo* fluorescence imaging allows the visualization of drug uptake (Fig. 5a). The increase of fluorescence with HT duration for thermistor and laser heating confirms the observations by the computer model (Fig. 3) and is supportive of earlier studies that demonstrated increased drug delivery with heating duration for TSL-DOX [1], [22], [24]. Similarly, the results for water bath HT agree with the computer model and show no significant difference in fluorescence between 15 and 60 min HT (Fig. 5(a),(b)). Based on these results, thermistor and laser provided significantly more effective tumor drug delivery, particularly at the longer 60 min HT duration.

### E. EXCISED TUMOR FLUORESCENCE IMAGING VISUALIZES TUMOR DOX DISTRIBUTION

**FIGURE 6.**
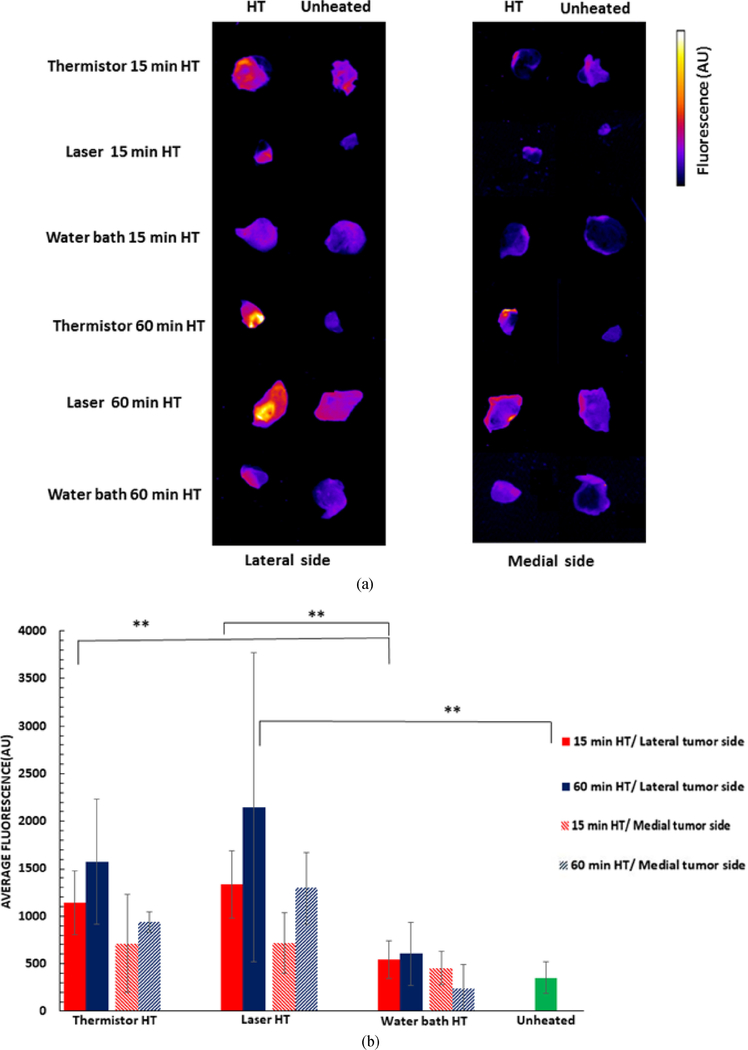
(a) Tumor fluorescence in representative samples of excised tumors for the three heating modalities (15 and 60 min HT duration) demonstrate differences in DOX uptake. Both lateral (i.e., tumor side facing the heating device) and medial side images are shown. (b) Average excised tumor fluorescence of all animal tumors. Error bars indicate standard deviation, and significant difference between groups is indicated: ∗∗(p *<* 0.001).

The drug uptake in excised tumors was visualized by fluorescence imaging from both the lateral (i.e., side facing the heating device) and the medial side (i.e., side distal from the heating device) (Fig. 6). For both laser and thermistor, the heated tumor fluorescence was either significantly higher than the unheated tumor, or approaching significance, for both lateral and medial sides (Table S5, Suppl. Mat.). There was no significant difference between heated and unheated tumors for water bath heating. In general, it appeared that more drug was delivered to the lateral side facing the heating device for all methods (Fig. 6), though this difference did not reach statistical significance in most cases (Table S5, Suppl. Mat.). Compared to the unheated control tumors, after 15 min HT, tumor fluorescence (mean of lateral and medial) was increased by 2.6× (thermistor), 2.9× (laser), and 1.4× (water bath); after 60 min HT, the increase was by 3.6× (thermistor), 4.9× (laser), and 1.2× (water bath) (Fig. 6(b)). Both thermistor and laser delivered significantly more drug to tumors (both to lateral and medial sides) than the water bath, but there was no significant difference between thermistor and laser. Unlike for surface fluorescence measurements, there was no statistically significant difference between 15- and 60-min HT for any heating modality, though a trend for higher drug uptake at 60 min HT was apparent for thermistor and laser (Fig. 6(b)). Overall, these results are in agreement with the computer model results, though the *in vivo* study did not clearly establish the benefit of laser over thermistor, even though there was a trend towards higher tumor drug uptake with laser (Fig. 6(b)).

### F. PLASMA PHARMACOKINETICS OF TSL-DOX IN VIVO AND IN COMPUTER MODELS

**FIGURE 7.**
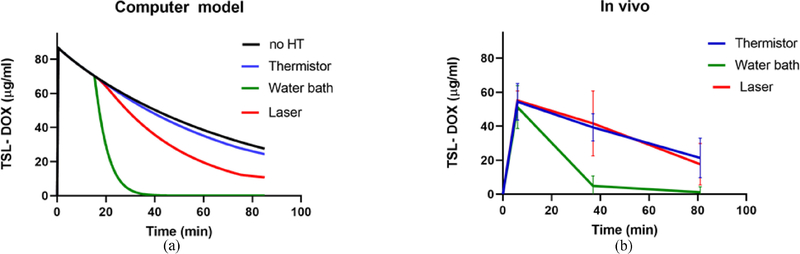
Plasma pharmacokinetics (PK) of TSL-DOX for each heating modality, shown (a) for computer model results, and (b) for in vivo studies. Plasma concentration in vivo was significantly lower for water bath compared to both laser and thermistor, at both 40 min and 80 min (p *<* 0.001). The half-life of TSL-DOX in the computer model was 51 min (no HT), which is similar to the half-life of 56 min in a prior study in this same animal model without heating [1].

Since the computer model included a systemic plasma compartment, plasma pharmacokinetics (PK) of TSL-DOX could be analyzed. Based on plasma PK in the *in vivo* studies from extracted plasma samples, we could make a direct comparison to computer model results (Fig. 7). In addition, in the computer model we show a case without heating, showing the PK of TSL-DOX with a half-life of 51 min due to leakage, clearance, and elimination. When localized HT is applied, the DOX release from TSL in the heated region results in an additional decrease of systemic TSL-DOX. In the computer models, this additional decrease was small to moderate for the more focused heating methods of thermistor and laser, (Fig. 7(a)). The plasma concentration of TSL-DOX was 85 *μ*g/g at the start of HT; after 15 min, this concentration decreased to 57.5 *μ*g/g without heating, to 56.1 *μ*g/g after thermistor HT, and to 48.2 *μ*g/g after laser HT. In stark contrast, plasma concentration of TSL-DOX decreased to 3.2 *μ*g/g after 15 min of water bath HT. A similar pattern was observed in the *in vivo* studies (Fig. 7(b)), though here we did not have a group without HT. In the *in vivo* studies, there was no significant difference between thermistor and laser PK, but the plasma concentration of TSL-DOX after water bath heating was again significantly lower than for either laser or thermistor (Table S13–14, Suppl. Mat.). These results confirm that the rapid depletion of systemically available TSL-DOX is responsible for the poor drug delivery efficacy from water bath heating and explains why the extension of the HT duration from 15 to 60 min did not provide a benefit.

## III. DISCUSSION

Thermosensitive liposomes (TSL) are stimuli-responsive drug delivery systems that release the encapsulated agent upon exposure to hyperthermia (HT) (T *>* 40 °C). The first known TSL formulation was developed several decades ago, and released within minutes [11]. Many of the more recent TSL formulations release the drug within seconds and are based on the intravascular triggered drug delivery paradigm, where drug release occurs within the tumor vasculature, followed by rapid tissue uptake of the released drug [12], [14], [25]. Known factors that affect tumor drug uptake with this intravascular-triggered TSL delivery approach include tumor temperature, the duration of HT [1], [22], [24], and core body temperature (as it affects systemic drug leakage) [1]. In the present study, we employed a rapid-release formulation of TSL-encapsulated doxorubicin (TSL-DOX) originally developed by Needham *et al.* [26]. We developed 3-D computer models based on the accurate geometry of a mouse hind limb for the preclinical evaluation of three different HT methods (i.e., thermistor probe, infrared laser, and water bath) on TSL-based drug delivery, with two heating durations (15-min and 60-min HT). Prior studies have employed HT durations as low as 2 min, with 60 min on the upper end [1], [14], [15]. Since longer HT enhances drug release and delivery for TSL [1], [22], [24], we employed two HT durations: 60 min to maximize delivery, and 15 min to evaluate the impact of a reduced HT duration. We furthermore carried out comparative *in vivo* studies where we used fluorescence imaging to monitor tumor drug uptake of the naturally fluorescent DOX [1]. The goal was to use the computer model to explain any observed differences in drug delivery and validate the computer model by the *in vivo* studies. All three heating methods achieved similar tumor surface temperatures, in the range of 42–43 °C (Fig. 2(a)–(c)).

Thermistor and laser achieved very localized heating of the tumor (Fig. 2(a), (b)), and as result produced localized drug delivery (Fig. 2(d), (e)). The surface temperature during thermistor heating was in agreement with a prior *in vivo* study where this thermistor probe with same heating parameters was used [1]. In contrast to thermistor and laser, the water bath produced uniform heating of the entire hind limb (Fig. 2(c)), and as result DOX delivery also occurred throughout the limb (Fig. 2(f)), but with reduced tumor drug uptake compared to laser or thermistor. When HT duration was extended from 15 to 60 min, thermistor and laser heating achieved further enhanced tumor drug uptake as anticipated based on prior studies [1], [22], [24], while water bath heating did not produce any enhanced tumor drug delivery (Fig. 2, and 3).

These observations based on the computer model were confirmed by whole-body *in vivo* fluorescence imaging studies in a mouse tumor model, where the same HT methods were employed after TSL-DOX administration. Both thermistor and laser showed increased localized drug uptake compared to the water bath after both 15 and 60 min HT, indicated by significantly higher *in vivo* tumor fluorescence (Fig. 5(a)). The water bath caused uniform delivery throughout the mouse hind limb since the entire limb was immersed in heated water. Also, in agreement with the computer models, tumor drug uptake increased after 60 min compared to 15 min HT for thermistor and laser, but not for water bath heating (Fig. 5(b)).

Effective cancer therapy requires adequate drug penetration throughout the whole tumor, and we examined this penetration via the computer model. The use of a 3-D computer model allowed us visualization of temperature, tumor drug penetration and cell survival fraction for the three heating modalities in a slice centrally through the tumor (Fig. 3). While both thermistor and laser produced focused heating at the tumor surface, for thermistor heating regions distal from the surface (*>*15 mm) experienced a significant reduction in drug delivery owing to inadequate temperatures. In contrast, the laser provided a considerably deeper heat penetration with most tumor regions obtaining temperatures in the optimal range for TSL-DOX release (*>*40 °C), resulting in deeper drug penetration from the heated surface (Fig. 3(e), (h)). The water bath produced uniform temperatures and drug uptake throughout the tumor and the remainder of the limb, but at much lower concentrations than thermistor or laser (Fig. 3(c), (f),(i)). The relative drug distribution remained similar for 60 min HT. But again, thermistor and laser produced higher tumor drug uptake with longer HT duration whereas for water bath HT there was no change in tumor drug uptake when HT duration was extended. Predicted tumor cell survival fraction was best for 60 min laser HT (Fig. 3(n)), with likely inadequate cell kill for the other HT conditions (Table I). For all three heating modalities, steady state temperature was obtained after 3–8 min, i.e., the enhanced uptake observed for 60 min HT was not due to any changes in tumor temperature beyond 15 min HT. While reduced tumor drug delivery with water bath HT compared to laser for TSL-DOX has also been demonstrated in a prior *in vivo* study, the cause was not established in this prior study [18].

The fluorescence measurements in extracted tumors generally support the results above (Fig. 6). Not unexpectedly, there was a trend towards larger drug uptake to the lateral tumor side (proximal to the heating device) than the medial tumor side, but only for thermistor at 60 min HT and for laser at 15 min HT was the difference significant (Fig. 6(b)). In the majority of comparisons for laser and thermistor HT, the tumor fluorescence was significantly higher in heated than in unheated control tumors. However, no significant difference was observed in any comparison of heated vs. control tumors for water bath heating (Table S5, Suppl. Mat.).

We further utilized the computer models to examine transport kinetics at the cellular level, i.e., transport of released DOX between tumor plasma, interstitium/extravascular extracellular space (EES), and intracellular tumor compartments. We selected the central location at the tumor surface to compare this drug transport kinetics by plotting drug concentration in each compartment over time (Fig. 4). In all cases, the majority of the drug after completion of HT was intracellular, which is the intended target compartment. This has been observed in prior intravital microscopy studies as well [25]. Compared to water bath HT, higher intracellular DOX concentrations were observed with the focal heating methods thermistor and laser, which were further increased when HT duration was extended. The water bath resulted in a lower cellular DOX concentration than the two other HT methods for both HT durations, and changed little when HT duration was increased (Fig. 4(c), (f)).

The analysis of the drug transport kinetics also explains the reason for the reduced efficacy of water bath HT: the heating of the entire hind limb causes DOX release from TSL in the entire limb, i.e., in a large tissue volume compared to the body volume. The result of this untargeted drug release within a large tissue volume is a rapid depletion of available encapsulated TSL-DOX in systemic plasma (Fig. 4(c)). Since the amount of encapsulated TSL-DOX in systemic plasma represents drug available for release once entering the tumor vasculature, the result is reduced drug release and a reduced tissue uptake. With water bath heating, this systemic drug reservoir (i.e., liposomal TSL-DOX in the systemic plasma) was almost completely depleted after only 15 min HT (Fig. 4(c)). Therefore, an increase in HT duration to 60 min provided no therapeutic benefit in the case of water bath HT (Fig. 4(f)). This conclusion is supported by pharmacokinetic (PK) measurements from the *in vivo* studies with the three HT methods. Also *in vivo*, only negligible amounts of TSL-DOX were present in plasma samples after 15 min of water bath HT, in agreement with the computer models (Fig. 7). While the computer model also suggests a small difference in plasma PK of TSL-DOX between laser and thermistor (again due to the larger tissue volume heated by laser) such a difference was not detectable in the *in vivo* PK data.

Our results demonstrate the importance of targeted tumor heating in preclinical studies, where often larger volumes than necessary are exposed to HT. The large tumor size relative to body size in mice compared to humans is an additional contributing factor. Further, the results have clinical relevance as various HT devices such as microwave-, or focused ultrasound-HT are employed in combination with TSL-DOX clinically in human patients [9], [17]. And in some cases - such as for treatment soft tissue sarcoma or for chest wall recurrences after breast cancer - large tissue volumes may be exposed to HT clinically, which may deplete available TSL-DOX - similarly to the presented preclinical results. This study also provides some helpful information for future preclinical studies with TSL, and we list a summary of preclinical study guidelines in the supplementary materials.

One issue not considered in this study is that HT by itself is an effective chemosensitizer [27]. Thus, extending the HT duration may provide therapeutic benefits even if it does enhance drug delivery from TSL. A limitation of the current study is that we did not measure tumor drug concentration with quantitative methods, though a prior study demonstrated good correlation between tumor fluorescence as used here and tumor drug concentration [1]. Further improvement in computer model accuracy may be achieved if accurate model parameters are available for specific tumors, ideally measured *in vivo* in the same tumor model used for validation.

## IV. CONCLUSION

In summary, our results suggest that the infrared laser is the preferred preclinical heating modality of the three methods investigated here. Importantly, the study concluded that water bath HT – the most widely used HT method in rodents due to availability and simplicity of use – is a poor choice for effective drug delivery with TSL. The results have relevance for treatment of human patients, as also clinically a variety of heating devices with varying temperature profiles and heating volumes are employed in combination with TSL-DOX. We demonstrated that computer modeling can aid the development of better drug delivery methods and can help provide explanations for differences in tumor drug uptake observed *in vivo*. While here the computer models were applied for preclinical studies, similar models could be developed for human patients to aid in the development of more effective cancer therapies based on TSL-mediated drug delivery.

## V. MATERIALS AND METHODS

### A. COMPUTER MODELS

#### 1) DEVELOPMENT OF THE MODEL GEOMETRY

A three- dimensional (3-D) handheld scanner was used to scan a nude mouse. Slow 360° scans were carried out to obtain a 3-D profile of the hind limb (Fig. 1(a)). After post-processing the 3-D data (Meshlab 2016.12.2, Autodesk Meshmixer 3.5), the resultant hind limb geometry was imported into computer modeling software (Comsol Multiphysics, 5.3). Subsequently, an ellipsoid was created embedded in the hind limb to represent a tumor (5.6 × 5.2 × 3.2 mm^3^ = 93.2 mm^3^, protruding 0.8 mm from the skin).

#### 2) MATHEMATICAL FORMULATION

Two computer models were coupled: (1) a heat transfer model to simulate tissue heating, and (2) a drug delivery model to simulate drug transport kinetics. The models were simulated in 3-D and the finite element method was used to solve the model equations (Comsol Multiphysics, 5.3). Heat transfer was modeled using Pennes’ bioheat transfer equation [28] with temperature dependent changes in perfusion, and tissue heating was simulated via three HT methods: (1) thermistor probe, (2) water bath, and (3) laser (as seen in Fig. 1(a)). Each HT (HT) method (42–43 °C target temperature) was applied for either 15 or 60 min. Temperature and perfusion from the heat-transfer model served as input variables for the drug delivery model that simulated the release of doxorubicin (DOX) from temperature sensitive liposomes (TSL) (after administration of TSL-DOX @ 5 mg/kg), transvascular transport of released DOX, and intracellular drug uptake by the tumor cells (Fig. 1(b)). Hind limb muscle and tumor had individual tissue properties assigned (Table S2, Suppl. Mat.). The rate of DOX release from TSL within the plasma was dictated by local temperature based on *in vitro* release measurements (Fig. S2) [21], and tumor cell survival fraction was estimated based on *in vitro* data in the same cell line (Fig. S1)[23]. The detailed description of the model assumptions and equations for the heat transfer and drug delivery models are available in the supplementary text.

### B. EXPERIMENTAL STUDIES

#### 1) THERMOSENSITIVE LIPOSOME PREPARATION

Thermosensitive liposomal doxorubicin (TSL-DOX) was prepared as per the protocol reported in an earlier published study [1]. Lipids dipalmitoyl-sn-glycero-3-phosphocholine (DPPC), monostearoyl phosphatidylcholine (MSPC) and 12-distearoyl-sn-glycero-3-phosphoethanolamine-NPEG2000 (DSPE-PEG2000) (Avanti Lipids, Alabaster, AL, USA) were dissolved in chloroform at a molar ratio of 85.7:9.7:5.0 (DPPC:MSPC:DSPE-PEG2000), then dried in flowing air to form a thin film of lipids. 300 mM citrate buffer (pH 4.0) was added to this lipid film and then kept in a water bath at 55 °C for 1 hour. An hour later, this mixture was extruded 5x through a 100 nm filter in a thermobarrell extruder (Lipex, Northern Lipids, Canada) at 60 °C. Dissolution of DOX hydrochloride (Sigma-Aldrich, USA) was carried out in deionized water (2 mg/mL) and loaded into TSL by pH gradient with phosphate buffered saline (PBS, pH 7.4).

The release kinetics of TSL was measured between 37 °C and 45 °C with a microfluidic device [29]. Briefly, TSL- DOX was diluted to 80 *μ*g/ml with the help of PBS at room temperature. This solution was then pumped through a millifluidic device, where a thin capillary tube was pre-heated to a pre-defined temperature while performing fluorescent imaging of the capillary (In vivo Extreme imaging system, Bruker, MA, US). As TSL-DOX passes through the tube, DOX is released. Since DOX fluorescence is quenched while encapsulated, the release can be detected as an increase in fluorescence, from which the released fraction is calculated [29]. The liposomal particle size was measured through dynamic light scattering (Zeta View Nanoparticle Tracking Analyzer (NTA), Mebane, NC, US)).

#### 2) PLASMA DOX QUANTIFICATION

The plasma concentration of DOX was carried out as described earlier [1]. The time of blood collection for PK estimation was 6, 37 and 81 min. Briefly, 30 *μ*l of sample plasma was added to 90 *μ*l of phosphate buffered saline (PBS) and 100 *μ*l of 10% Triton X-100 (diluted in deionized water). The fluorescence of the sample plasma was determined through a microplate reader (Synergy HT, Biotek Instruments Inc., Winooski, VT, USA) using suitable filters for DOX (excitation 485 nm, emission 590 nm). DOX concentration was determined based on a standard curve prepared from clean mouse plasma mixed with known concentrations of DOX (1–100 *μ*g/ml).

#### 3) TUMOR MODEL

Lewis lung carcinoma cells (LLC1, RRID: CVCL_4358) were authenticated by short-tandem repeat (STR) profiling (LabCorp, Sept. 28th 2020), and tested for mycoplasma contamination. The cells were routinely maintained at 37 °C in 5% CO2/95% air in Dulbecco’s modified Eagle’s medium (Sigma-Aldrich, D6429) enhanced with 10% inactivated fetal bovine serum (Invitrogen), 1% Penicillin and streptomycin (Invitrogen). Once these cells reached confluence, the cells were trypsinized, and centrifuged to obtain a pellet. The cell pellet was then suspended in Hank’s balanced salt solution (HBSS) and reconstituted to achieve 1 × 10^6^ cells per injected site. These cells were injected in both the hind limbs of NCI Athymic NCr-nu/nu mice (8–12-week-old, female), and tumors were grown until ~4–5 mm size. Sex as biological variable was not considered, as the focus of this *in vivo* study was to validate a computer model that did not include any sex dependent parameters. A routine screen was employed for cells in culture to avoid any contamination. All the experiments were approved by the Medical University of South Carolina’s Institutional Animal Care and Use Committee (Protocol# AR 18–00385).

#### 4) IN VIVO STUDIES

Mice (n = 4 per group) were injected with TSL-DOX at a dose of 5 mg/kg. 15 min after injection, one of the two tumors was heated with one of the three HT methods, for either 15 min or 60 min. While ideally, HT should start immediately after injection to maximize tumor drug uptake (see Fig. S4), the 15 min delay was required to obtain a blood sample, perform imaging, and set up the HT device following TSL-DOX administration. The tumor (left or right), hyperthermia (HT) method and HT duration were randomly selected. Contralateral, unheated tumors served as control in each of the animals. Three HT methods were employed: (1) For thermistor probe heating, a customized probe was fabricated on the basis of a 2.5 mm diameter thermistor (Honeywell, NTC 121) [1]. (2) For water bath HT, a temperature-controlled laboratory water bath was heated to 42 °C. A customized animal tray was designed so that only the mouse hind limb carrying the tumor was immersed in the heated water. The tray was thermally insulated to avoid heating of the mouse body. Petroleum jelly was applied to the hind limb before immersing it in water to prevent swelling. (3) The third HT method was an infrared laser (1 W, 850 nm wavelength). A thermocouple placed on the skin surface was employed to keep temperature at 43 °C by adjusting the applied laser power. Fluorescence imaging was employed to quantify tumor drug uptake. Whole-body fluorescence imaging of the mice was carried out before TSL-DOX injection, after injection, and after tumor HT by an *in vivo* imaging system (In vivo Extreme imaging system, Bruker, MA, US). After imaging, mice were sacrificed, and the excised tumors were imaged from both lateral and the medial sides. For imaging of DOX fluorescence excitation and emission filter wavelengths were 550 nm and 600 nm, respectively.

### C. STATISTICAL ANALYSIS

A generalized linear regression model for repeated measures was used to make the following comparisons for the different treatment groups: heated vs. control tumor; thermistor vs. laser vs. water bath; 15 vs. 60 min HT. Least-square means were compared by two-sided t-tests and the level of significance was held at *α* = 0.05. All statistical analyses were performed in SAS 9.4 (SAS Institute, Cary, NC). Post-hoc power analysis using a two-sided t-test was done for comparisons of in vivo fluorescence measurements of heated tumors between the three heating methods, for 15 and 60 min HT. Power analyses were performed in PASS 2008 (NCSS, Kaysville, UT).

## Supplementary Material

supp1-3078843

## Figures and Tables

**TABLE I:** Tumor Drug Concentration and Survival Fraction for Different HT Methods

Hyperthermia (HT) method + duration	Tumor Drug Concentration (μg/g)	Tumor Survival Fraction (%)
	Mean [Range]	Mean [Range]

Thermistor 15 min	8.8 [0.3–18.5]	53.7[12.8–100]
Thermistor 60 min	21 [0.3–36.8]	11.6[0.78–100]
Laser 15 min	14.1 [9.9–17.5]	22.7[14.5–39.1]
Laser 60 min	25.2 [19.7–28.9]	3.7[2–7.9]
Water bath 15 min	9.4 [9.1–9.6]	41.5[40.1–42.4]
Water bath 60 min	8.7 [8.2–8.9]	39.6[38.8–40.6]
no HT	0.68 [0.66–0.69]	100

## IMPACT STATEMENT

Untargeted large volume hyperthermia (HT) rapidly depletes systemically available thermosensitive liposome-encapsulated drug, resulting in low tumor drug uptake. Computer models established the cause for the observed differences in tumor drug uptake for varying HT methods.
