# Supplementary material for: Untargeted Large Volume Hyperthermia Reduces Tumor Drug Uptake From Thermosensitive Liposomes
Source: IEEE Open J Eng Med Biol. 2021 May 11;2:187–97. doi: 10.1109/OJEMB.2021.3078843 (PMC8562592; doi:10.1109/OJEMB.2021.3078843)
Supplement: Supplementary Materials: Untargeted Large Volume Hyperthermia Reduces Tumor Drug Uptake From Thermosensitive Liposomes [file supp1-3078843.pdf]

Supplementary Materials

Untargeted Large Volume Hyperthermia Reduces Tumor Drug Uptake From Thermosensitive Liposomes

Krishna K. Ramajayam[1], A. Marissa Wolfe[2], Anjan Motamarry[1], Georges J. Nahhas[3,4], JohnYost[5], Michael J. Yost[5], and Dieter Haemmerich[1*] *Senior Member, IEEE*

[1]Department of Pediatrics, Medical University of South Carolina, Charleston, SC 29425
[2]Ralph H. Johnson VA Medical Center, Charleston, SC 29401
[3]Hollings Cancer Center, Medical University of South Carolina, Charleston, SC 29425
[4]Department of Psychiatry and Behavioral Sciences, Medical University of South Carolina, Charleston, SC 29425
[5]Department of Surgery, Medical University of South Carolina, Charleston, SC 29425

CORRESPONDING AUTHOR: Dieter Haemmerich (e-mail: haemmer@musc.edu)

This work was supported by NIH Grant Number RO1CA181664. Part of this work was conducted in a facility constructed with support from the National Institutes of Health, C06 RR015455 and Grant Number C06 RR018823. This article has supplementary downloadable material

I. Methods

A. Computational Models

1) Development of the Model Geometry

A three-dimensional (3D) handheld scanner was used to scan a nude mouse. Slow 360˚ scans were carried out to obtain a 3D profile of the hind limb (Fig. 1(a) in main manuscript). Subsequently, the following steps were taken to refine the hind limb geometry: 1) Duplicate faces, vertices and isolated pieces of the scanned geometry were removed, unreferenced vertices were checked for, and close vertices were merged using MeshLab v2016.12; 2) The geometry was examined for remaining gaps, and mesh density was optimized in Autodesk Meshmixer. The mesh was then imported into a finite element modelling software (COMSOL Multiphysics, 5.3). An ellipsoid geometry was then created in the hind limb to depict the tumor.

2) Grid independency

The final model employed a composite mesh where the tumor had a more fine mesh than the other domains. For verifying the effect of mesh size on the model results, the mesh size of all model domains was increased by one level (coarse mesh), and decreased one level (fine mesh). The following parameters were used in the respective grid settings:

Table S1. Grid independency parameters.

Parameter	Course mesh	Fine mesh	Default mesh
Max element size	6.53 mm	3.48 mm	4.35 mm
Min element size	1.22 mm	0.43 mm	0.78 mm
Max element growth rate	1.6	1.45	1.5
No of elements	7603	34836	16544

Compared to the default mesh, the average tumor temperature varied by 0.05 ˚C (course mesh) and 0.002˚C (fine mesh). The average tumor DOX concentration varied by 0.23 µg/ml (course mesh) and 0.02 µg/ml (fine mesh). These results demonstrate that the employed default mesh was adequate.

3) Mathematical Model Formulation

The mathematical formulation was developed based on a coupled heat transfer and drug delivery model simulated in a three-dimensional volume. A finite element approach was used to solve this formulation in the software COMSOL Multiphysics, 5.3. Tissue heat transfer and drug delivery equations were solved via a multifrontal massively parallel sparse direct solver (MUMPS) employing a segregated solution approach [30].

4) Heat Transfer Model

The heat transfer model simulated tissue heating via three hyperthermia methods, namely; thermistor probe (a customized heating probe), water bath and infrared (IR) laser, for both 15- and 60-min HT. Perfusion (perfusionDec) was varied with the degree of stasis as per an earlier published study, where vascular damage was calculated based on temperature history via an Arrhenius damage model [1]. The Arrhenius parameters and the relationship between vascular damage and perfusion were different for normal and for tumor tissue. For normal tissue an initial increase in perfusion was considered followed by stasis [1], whereas for tumor no initial increase was considered based on prior studies [2, 3].

a) Thermistor Heating Probe

The thermistor heating probe was based on a thermistor bead (2.5 mm diameter) that was placed in contact with the tumor surface (Fig. 1a). The bead was modeled as two concentric spheres as described in a prior study (25). The inner sphere (lead telluride, C_p = 159 J/(kg K), k = 0.1 W/(m.K), ρ = 6300 kg/m^3) represents a distributed heat source with power adjusted to obtain a temperature of 50˚C. The outer shell (0.37 mm thickness) was made of silica glass (C_p = 703 J/(kg K), k

= 1.38 W/(m K), ρ = 2203 kg/m^3). The upper and lower surfaces of the mouse hind limb were cooled due to convective cooling of air at 25˚C (normal ambient room temperature). The calculation of the convective heat transfer coefficient h was based on approximations for convective cooling of an upward-facing horizontal plane (for the upper leg surface) and a downward-facing horizontal plane (for the lower leg surface), with following parameters: plane perimeter = 0.12 m, plane area = 0.0005 m^2, film temperature = 31°C, ΔT = 12°C, all thermophysical properties of air were obtained at film temperature [4]. The values for h were thus determined to be 10.1 and 5 W/(m^2 K) for upper and lower surface of the hind limb, respectively. The input power to the thermistor was adjusted such that the surface temperature was between 42-43˚C.

b) Water Bath Heating

In this case, the entire mouse hind limb with the tumor geometry was immersed in water at 42.5-43˚C with heat flux boundary condition and h = 554 W/m^{2}K. We calculated h considering the approximation for a horizontal cylinder with following parameters: cylinder length = 0.045 m, cylinder diameter = 0.025 m, film temperature = 39.5°C, ΔT = 5°C, all thermophysical properties of water were used at film temperature [4].

c) Infrared Laser Heating

Laser light (850 nm) was applied to the tumor in x-direction. Laser heating was modeled by a distributed heat source with a Gaussian profile f(y,z) orthogonal to the laser light direction [5]. Absorption and scattering along the direction of light propagation (i.e. x-axis) were considered according to Equ. 1. The laser power Q_o was set constant such that the tumor surface temperature reached 43˚C. Convective cooling of ambient air around the hind limb surface was modeled as described above for the thermistor heating probe.

The equation that represents the laser input power is shown below:

$$\mathrm{Q} = \mathrm{Q_0} \cdot (1 - \mathrm{R_c}) \cdot \mathrm{A_c}\left(\frac{1}{\pi\sigma_z\,\sigma_y}\right) \cdot \exp\left(-\left(\frac{(z-zo)^2}{2\sigma_z{}^2}\right) - \left(\frac{(y-yo)^2}{2\sigma_y{}^2}\right)\right) \cdot \exp(\mathrm{u_s} + \mathrm{A_c}) \cdot \mathrm{abs}(\mathrm{x} - \mathrm{xo}) \qquad (1)$$

The modelling of heat transfer in the hind limb was carried out using Pennes' bio-heat transfer equation (24), with temperature dependent changes in perfusion:

$$\rho C \frac{\partial T}{\partial t} = \nabla \cdot k\nabla T + Q_H - Q_P + BMR \qquad (2)$$

where ρ is tissue density; C is specific heat of tissue; k is tissue thermal conductivity, T is spatially and temporally varying tissue temperature, Q_H represents the heat source (depending on hyperthermia method: thermistor, water bath or laser); $Q_P=\rho_{\text{blood}}{*}C_{\text{blood}}{*}F{*}(T\text{-}T_{\text{blood}})$ is the heat loss due to blood perfusion; and BMR=9815.6 W/m^3 [6] is the basal metabolic rate. The target tissue temperature achieved for hyperthermia treatment was 42˚C for all computational as well as *in vivo* studies. Table S2 shows the various parameters used in the computer model. In addition, Table S3 provides parameters and variables with specific values for tumor and muscle regions.

5) Drug Delivery Model

The outputs of temperature and perfusion from the heat-transfer model were fed as inputs to the coupled drug delivery model that simulated the release of doxorubicin from temperature sensitive liposomes (TSL) inside vasculature, transvascular transport of released doxorubicin into interstitium, and intracellular drug uptake by the tumor and muscle cells. Figure 1(b) (main text of manuscript) provides an overview of the drug delivery model. The mouse hind limb had two individual regions for muscle tissue and tumor, each with their individual properties (16). Cellular uptake was based on *in vitro* data of DOX uptake by Lewis lung carcinoma (LLC) cells, considering both passive and active uptake mechanisms [7]; for muscle, the same cell uptake model was employed as no data for muscle cells was available.

6) Equations

The following ordinary differential equations (ODEs) were utilized to describe the drug delivery model. In equations 3-7, several variables and parameters are specific to tissue region (tumor or muscle), and will therefore carry the superscript T or M (superscripts are omitted in the equations, but are listed in Table S3). For example, F_{pv} in equation 4 will correspond to either $F_{pv}{}^{T}$ (in tumor) or $F_{pv}{}^{M}$(in muscle).

The concentration of the liposomal encapsulated drug in the systemic plasma is as follows:

$$\frac{dc_{p_{Lip}}(x,y,z)}{dt} = \frac{D/V_p{}^B}{T_{inf}} - k_{e_{Lip}} \cdot c_{P_{Lip}} - \int_V R.c_{p_{Lip}} dV \cdot \frac{v_p}{V_p{}^B} \qquad ; t \le T_{inf}$$

$$\frac{dc_{p_{Lip}}(x,y,z)}{dt} = -k_{e_{Lip}} \cdot c_{P_{Lip}} - \int_V R.c_{p_{Lip}} dV \cdot \frac{v_p}{V_p{}^B} \qquad ; t > T_{inf}$$

(3)

where the first term on the right side of the upper equation describes the infusion of the drug (i.e. this term is equal to zero at t>T$_{\text{inf}}$, and thus omitted in the lower equation). The second term describes the clearance, and the third term accounts for the release of the DOX from the liposomes due to heating. The volume V represents the integration volume and includes the whole hindleg, including both tumor and muscle regions.

The locally varying **concentration of unencapsulated DOX in tumor plasma of the hind limb** was modeled per following equations (for tumor, and muscle tissue).

$$\frac{dC_p\ (x,y,z)}{dt} = -\frac{1}{v_p}\cdot PS(c_p - c_e) - F_{pv}\cdot c_p + F_{pv}\cdot c_p^B + c_{p\_Lip}.R \qquad (4)$$

The first term on the right describes drug transport between plasma and interstitium. While transport processes other than diffusion could potentially contribute to transvascular transport (e.g. convection), the modeling approach assuming diffusion transport is appropriate as long as net transport can be represented accurately by an apparent permeability, and this method has been employed in numerous prior studies [8-10]. Furthermore, for small molecules such chemotherapeutic drugs, diffusive transport is typically dominating over convection due to the comparably high diffusivity of such agents [11]. The second and third right term represents the DOX entering and exiting the hind limb. The final term defines the intravascular release from TSL-DOX. The temperature dependent TSL release kinetics were modeled based on earlier published studies [10, 12].

Further, **the concentration of unencapsulated DOX in EES** was modeled per following equation:

$$\frac{dc_e\ (x,y,z)}{dt} = \frac{1}{v_e}PS(c_p - c_e) - k_{3ci}\left(k_{1ci}c_e + \frac{k_{2ci}c_e}{K_{ici}+c_e}\right) \qquad (5)$$

The first term on the right accounts for the transvascular transport of DOX between the plasma compartment and the EES, and the second term accounts for the decrease in the EES concentration due to DOX which is taken up by cells.

The **intracellular DOX concentration** was modelled per earlier published studies [27-29] according to following equation:

$$\frac{dc_i\ (x,y,z)}{dt} = k_{3ci}\left(k_{1ci}c_e + \frac{k_{2ci}c_e}{K_{ici}+c_e}\right) \qquad (6)$$

As mentioned, this cell uptake is based on *in vitro* studies with LLC cells. No separate uptake model was implemented for normal muscle tissue due to lack of data. However, since the focus of this study was the analysis of tumor drug uptake, this limitation is not of great significance here.

<u>Systemic Compartments</u>:

Systemic compartments for plasma and tissue were included without spatial dependence. The systemic plasma compartment concentration considers inflow and outflow from the hindlimb (first and second terms of equation (7)), as well as clearance and uptake by body tissue. **The concentration of unencapsulated drug in the systemic plasma compartment** was described by:

$$\frac{dc_p{}^B}{dt} = \Big(\int_V BV\cdot\frac{v_p}{V_p{}^B}(1-HctT)F_{pv}\cdot c_p\ \cdot dV) - c_p{}^B\int_V BV.\frac{v_p}{V_p{}^B}\cdot(1-HctT)\cdot F_{pv}\cdot dV) + R\cdot c_{p\_Lip}\frac{V_p{}^B}{VD} - k_e\cdot c_p{}^B - k_p\cdot c_p{}^B + k_t\cdot c_t{}^B\Big) \qquad (7)$$

The **DOX concentration in systemic tissue** was calculated by the following equation:

$$\frac{dc_t{}^B}{dt} = \left(k_p\cdot c_p{}^B - k_t\cdot c_t{}^B\right) \qquad (8)$$

The **total unencapsulated drug concentration calculated in the tumor surface and tumor cross section** was based on the volume fractions of tumor plasma ($v_p{}^T$), tumor interstitium ($v_e{}^T$), and tumor cells ($v_i{}^T$), and was used for visualization of drug concentration in figures 2 and 3 of the main text:

$$c = ((c_i{}^T + c_{ib}{}^T).\ v_i{}^T + c_e{}^T v_e{}^T + c_p{}^T.v_p{}^T) \qquad (9)$$

Survival fraction of cells due to drug exposure

A prior study reported *in vitro* results where LLC cells were exposed to varying extracellular concentrations of doxorubicin [28]. This prior study also measured cellular drug uptake, and reported the relationship between survival fraction of LLC cells and peak intracellular concentration. Based on these data, we fitted an exponential curve and integrated the resulting equation in the computational model to predict survival fraction in the tumor. We converted the intracellular concentration from (ng/10^5 cells) to (µg/ml) as required by our model based on an estimated cell volume of 10^{-9} mL [31]. The data from this prior study with fitted equation is shown in Fig. S1 below:

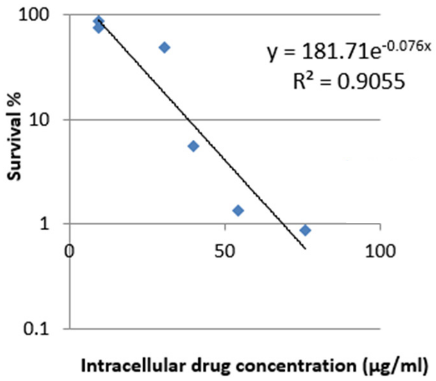

Fig. S1. Relationship between cancer cell survival fraction and intracellular DOX concentration from a prior study [28], with fitted exponential equation. To avoid survival predicted by the equation exceeding 100% at low intracellular concentrations, survival was limited to max. 100%.

Table S2. Complete list of model parameters

Symbol	Description	Value	Source
BW	Body weight for mice	20 g	Approximated from animal studies
VD	Volume of distribution	19e-6 m^3	Calculated with data from [13]
D	Total dose of encapsulated DOX injected	0.1 mg	5 mg/kg * bodyweight [14]
Hct	Hematocrit	0.45	[15]
Hct_{tumor}	Hematocrit for tumor microvasculature	0.19	[16]
k_p	Transfer constant from systemic plasma to lump tissue	9.4e-3 s^{-1}	[10]
k_e	Transfer constant for clearance	2.1e-3 s^{-1}	[10]
k_t	Transfer constant from tissue to systemic plasma	7.052e-5 s^{-1}	[10]
k_{e_Lip}	Rate constant of TSL clearance	2.228e-4 s^{-1}	fit to data from [17]
k_{1ci}	Parameter for intracellular uptake	2.257	[7]
k_{2ci}	Parameter for intracellular uptake	0.0452 kg/m^3	[7]
k_{3ci}	Parameter for intracellular uptake	2.806e-4 s^{-1}	[7]
K_{ici}	Parameter for intracellular uptake	5.29e-4 kg/m^3	[7]
PS	Permeability surface area product for DOX	0.011 s^{-1}	[18]
v_v^T	Tumor volume fraction of vascular space(1- v_i^T- v_e^T)	0.054	[19]
v_p^T	Tumor volume fraction of plasma space v_v^T *(1-HctT)	0.0437	assumed
v_e^T	Tumor volume fraction of EES	0.473	[20]
v_i^T	Tumor volume fraction of intracellular space	0.473	$(1-v_v^T-v_e^T)$
v_v^M	Muscle volume fraction of vascular space	0.04	[21]
v_p^M	Muscle volume fraction of plasma space v_v^M*(1-HctT)	0.0324	muscle plasma fraction (assumes same microvascular Hct as in tumor microvessels)
$R(T)$	Release rate of DOX from TSL	variable[s^{-1}]	[10]
ρ_{Tissue}	Mass density of tissue	1060 kg/m^3	[22]
BV	Blood Volume	0.06678	(6.3 g / 100 ml * ρ_{Tissue}) [23]
R_x	Universal gas constant for tissue damage model	8.31 J/molK	Universal value
$w0^T$	Tumor blood perfusion	0.0032 s^{-1}	[24]
$w0^M$	Muscle blood perfusion	0.003 s^{-1}	[22]
Q_o	Input power – Laser	0.2 W	Assumed
R_c	Optical reflection coefficient	0.1	[25]

Ac	Optical absorption coefficient	0.64 m^{-1}	[26]
σ_y	Laser width y direction	1 mm	Assumed
σ_z	Laser width z direction	1mm	Assumed
xo	Laser center x direction	12.2 mm	Assumed
yo	Laser center y direction	23.9 mm	Assumed
zo	Laser center z direction	399.9 mm	Assumed
u_s	Optical scatter coefficient	475 m^{-1}	[26]
ρ_{blood}	Mass density of blood	1000 kgm^{-3}	[10]
C_{blood}	Specific heat of blood	4180 J/kg	[10]
T_{blood}	Temperature of blood	37˚C	[10]
T_{inf}	Infusion time of TSL	30 s	Assumed

Table S3 Tumor and muscle parameters utilized in the computer model

**Symbol**	**Description**	**Value**	**Source**
Tumor			
F^T	Perfusion	$w0^T * perfusionDec^T$	[1]
$F_P{}^T$	Plasma perfusion	$w0^T * perfusionDec^T(1\text{-}Hct)$	Assumed
$F_{pv}{}^T$	Plasma Flow in tumor plasma space; note: F_{pv}=plasma flow/plasma volume	$w0^T * perfusionDec^T(1\ \text{-}Hct)/\ v_P{}^T$	Assumed
Ax	Arrhenius parameter for vascular damage	8.05e160 s^{-1}	[10]
EAx	Arrhenius parameter for vascular damage	9.96e5 s^{-1}	[10]
$perfusionDec^T$	Variable (range 0…1) that determines perfusion decrease based on Arrhenius model		[1]
Muscle			
F^M	Perfusion	$w0^M * perfusionDec^M$	[1]
$F_P{}^M$	Plasma perfusion	$w0^M * perfusionDec^M(1\text{-}Hct)$	Assumed
$F_{pv}{}^M$	Product of plasma perfusion and volume fraction	$w0^M * perfusionDec^M(1\ \text{-}Hct)/\ v_P{}^M$	Assumed
Ax	Arrhenius parameter for vascular damage	2e106 s^{-1}	[10]
EAx	Arrhenius parameter for vascular damage	6.7e s^{-1}	[10]
$perfusionDec^M$	Variable (range 0…1) that determines perfusion decrease based on Arrhenius model		[1]

II. Results

A. Characterization of Thermosensitive Liposomes (TSL)

1) TSL Release Kinetics

The release kinetics of TSL was determined by passing it through a heated microcapillary at different temperatures ranging from 37°C-45°C using a computer-controlled heat source. In the microcapillary set up, when temperature was greater than 40°C, substantial DOX release was observed from TSL. Furthemore, with increase in temperature to 45°C, maximal release was observed with approximately 80-85% of drug (DOX) released (Fig S2)

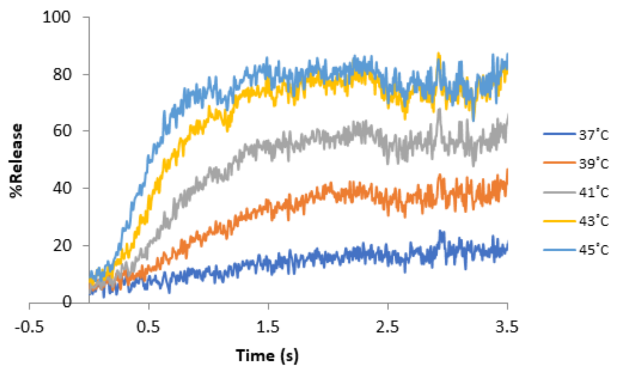

Fig. S2. Release kinetics of liposomal DOX at different temperatures

2) TSL Particle Size Distribution

Particle size distribution of the TSL was performed with the NTA Zetasizer system and the average particle size was determined to be 100.4±34.6 nm. This is well in agreement with our prior studies with these TSL [14].

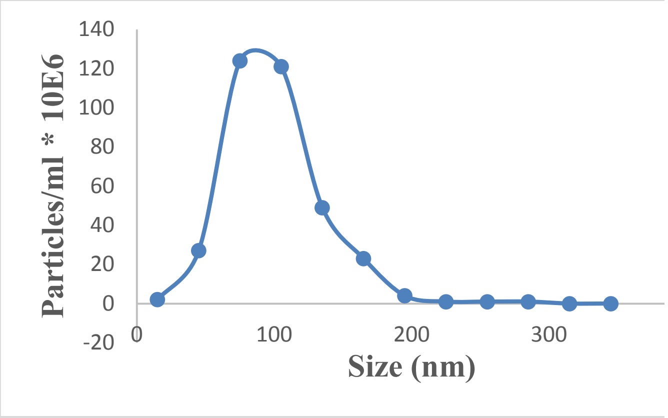

Fig. S3. Particle size distribution for TSL Release kinetics of liposomal DOX at different temperatures.

B. Plasma AUC Correlates with Fluorescence of Tumor ROI after Heating

Two prior studies demonstrated that the plasma-AUC of encapsulated drug calculated during hyperthermia is predictive of tissue drug uptake [12, 32]. This correlation can be explained by the intravascular triggered release paradigm on which these rapid-release TSL are based: TSL-encapsulated drug in systemic circulation continuously enters the heated tissue volume, releasing the drug. This process continues as long as heat is applied, and TSL-encapsulated drug is available in systemic circulation. Therefore, the longer heat is applied, the more drug is locally released. Since the plasma concentration of TSL-encapsulated drug represents the drug amount available for release, the AUC of plasma TSL-Dox concentration calculated during HT (Fig. S4a) is representative of the total amount of drug released in the heated volume during HT. This explains why this plasma AUC correlates with tissue drug uptake, where here tissue fluorescence serves as surrogate for concentration (Fig. S4b)

C. Guidelines for preclinical studies with TSL based on our results

Below we provide some guidelines based on our results for future preclinical studies in rodents, with the goal of achieving optimal drug delivery throughout tumors:

- Initiate hyperthermia either before administration of TSL, or as soon as practical after administration. This is to maximize the Plasma-AUC, which we found correlates well with tumor drug uptake (Fig. S4). Pre-heating is in particular advantageous in cases, when heating of the tumor requires some time (depending on hyperthermia method and heating device).
- Use a HT method that ensures heating of the whole tumor while avoiding exposure of normal tissues. To ensure adequate tumor heating, at minimum temperature at the distal edge of a subcutaneous tumor should be obtained to confirm that the whole tumor is exposed to hyperthermic temperatures where the employed TSL have optimal release (~40-43 ºC in our case). While MR thermometry is often not available or practical, such a method would be ideal to ensure targeted tumor heating.
- Obtain a blood sample after completion of HT, to ensure that available encapsulated drug has not been depleted (a comparison to a non-heated control group confirms that depletion is due to HT, rather than TSL leakage). If possible, a second blood sample after TSL administration and before HT would be valuable (e.g. allow estimating the plasma-AUC as in Fig. S4).
- While the required HT duration depends on many factors such as drug, tumor model, etc., in general, extending the HT duration enhances tumor drug uptake assuming encapsulated TSL is still in circulation.
- Provide optimal thermal support and monitor core temperature of animals during studies. Due to anesthesia, rodents are not able to regulate their core temperature and require thermal support. Prior studies have shown that elevated core temperature (>37 ºC) due to too much thermal support can result in premature drug leakage from TSL [14] while a reduced core temperature will make it more difficult to expose tumors to HT. Thus, ideally the core temperature should be regulated to ~36-37 ºC by adjusting thermal support as necessary.

D. Plasma PK in Computer Model and in vivo

After the administration of TSL-Dox, the plasma concentration in the computer model was 86 µg/ml and the half-life of TSL-DOX was 51 min, which is similar to an earlier published *in vivo* study [33] that reported a half-life of 56 min. Further details have been provided in Table S4.

E. Statistical Analysis Results

The supplementary Tables S5-S11 provides the statistical analysis results, where the listed values represent the mean fluorescence (AU) within a certain treatment group. There were six treatment groups, based on three HT methods (laser, thermistor and water bath), and two HT durations (15 min and 60 min). Each animal had two tumors, one tumor exposed to HT, and an unheated control tumor (CTRL). For each tumor there were three fluorescence measurements: tumor surface fluorescence measured by *in vivo* imaging after HT (SUR), as well as measurements in extracted tumors from lateral (LAT), and medial (MED) sides. Table S5 provides results of comparing HT and CTRL tumors in each treatment group. Table S6 and S7 shows results comparing the heating methods for 15 min HT (Table S6) and 60 min HT (Table S7). Tables S8-S9 compare 15 min with 60 min HT for laser (Table S8), thermistor (Table S9) and water bath (Table S10). Table S11 compares fluorescence of lateral (side from which heat was applied) and medial side of the excised tumors after HT in each group. Table S12 shows results of a post-hoc power analysis for *in vivo* imaging (SUR) for heated tumors (HT), comparing the different heating methods for 15 min and 60 min HT. Table S13 and S14 provide the statistical comparison for PK for 15 min and 60 min HT for various heating methods.

Table S4. Comparison of plasma PK (computer model vs *in vivo*)

		Plasma PK		
Time (min)	HT method	Computer Model (µg/ml)	*In vivo* (µg/ml)	Difference (µg/ml)
6	Thermistor	79.8	54.3	25.5
6	Laser	79.8	55	24.8
6	Water bath	79.8	51.5	28.3
37	Thermistor	50.3	39.2	11.1
37	Laser	39.1	41.6	2.5
37	Water bath	0.62	4.9	4.2
81	Thermistor	25.7	21.3	4.4
81	Laser	11.3	17.5	6.2
81	Water bath	0	1.1	1.1

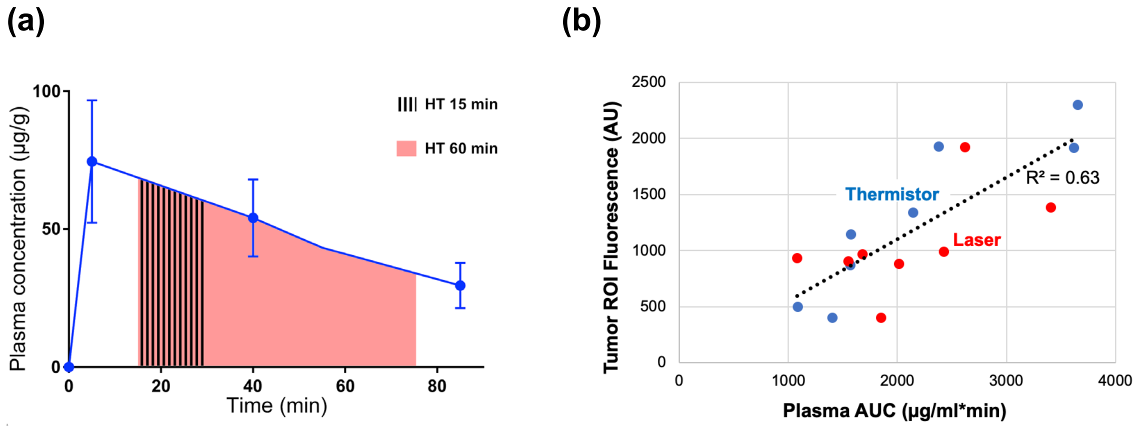

Fig. S4. Plasma-AUC correlates with tissue fluorescence. (a) Plasma-AUC of Dox concentration was calculated during heating, as indicated for either 15 or 60 min HT. (b) Plasma-AUC correlated well with fluorescence of the tumor ROI measured following HT (R^2=0.63). Data was pooled for thermistor and laser studies, including 15 min and 60 min HT data. Water bath data was excluded since no accurate estimation of the plasma AUC was possible due to the rapid decline in plasma concentration in water bath studies (see Fig. 7)

Table S5 HT vs CTRL: stratified by TREATMENT and TYPE.

Treatment	Type	CTRL	HT	CTRL - HT	*p*-value
T1-Laser-15 min	LAT	418	1336	-918	<.0001
T1-Laser-15 min	MED	508.75	716.75	-208	0.1542
T1-Laser-15 min	SUR	236.5	801	-564.5	<.0001
T2-Laser-60 min	LAT	660.02	2142.35	-1482.33	0.0574
T2-Laser-60 min	MED	309.35	1061.5	-752.15	0.002
T2-Laser-60 min	SUR	486	1294	-808	0.0002
T3-Thermistor-15 min	LAT	575	1140.5	-565.5	<.0001
T3-Thermistor-15 min	MED	352	711.75	-359.75	0.0275
T3-Thermistor-15 min	SUR	262.75	725.83	-463.08	0.015
T4-Thermistor-60 min	LAT	258.75	1572.75	-1314	<.0001
T4-Thermistor-60 min	MED	269.75	942	-672.25	<.0001
T4-Thermistor-60 min	SUR	277.75	1870.75	-1593	<.0001
T5-Waterbath-15 min	LAT	411	545.75	-134.75	0.2993
T5-Waterbath-15 min	MED	338.25	455	-116.75	0.1173
T5-Waterbath-15 min	SUR	230	409.75	-179.75	0.0003
T6-Waterbath-60 min	LAT	317	456.75	-139.75	0.195
T6-Waterbath-60 min	MED	177	182.25	-5.25	0.9656
T6-Waterbath-60 min	SUR	235.5	305.5	-70	0.6675

Table S6. TREATMENT (15 min) stratified by ARM and TYPE.

ARM	Type	T1-Laser-15 min	T3-Thermistor-15 min	T5-Waterbath-15 min	T1-T3	*p*-value	T1-T5	*p*-value	T3-T5	*p*-value
CTRL	LAT	418	575	411	-157	0.0591	7	0.9284	164	0.0296
CTRL	MED	508.75	352	338.25	156.75	0.472	170.5	0.2916	13.75	0.939
CTRL	SUR	236.5	262.75	230	-26.25	0.7102	6.5	0.945	32.75	0.7544
HT	LAT	1336	1140.5	545.75	195.5	0.3569	790.25	<.0001	594.75	0.0004
HT	MED	716.75	711.75	455	5	0.9848	261.75	0.0948	256.75	0.2772
HT	SUR	801	725.83	409.75	75.175	0.6904	391.25	0.0107	316.08	0.0777

Table S7. TREATMENT (60 min) stratified by ARM and TYPE.

ARM	Type	T2-Laser-60 min	T4-Thermistor-60 min	T6-Waterbath-60 min	T2-T4	*p*-value	T2-T6	*p*-value	T4-T6	*p*-value
CTRL	LAT	660.03	258.75	317	401.28	0.0001	343.03	0.0133	-58.25	0.5447
CTRL	MED	309.35	269.75	177	39.6	0.7072	132.35	0.2227	92.75	0.262
CTRL	SUR	486	277.75	235.5	208.25	0.1011	250.5	0.0684	42.25	0.7036
HT	LAT	2142.35	1572.75	456.75	569.6	0.4531	1685.6	0.0201	1116	0.0008
HT	MED	1061.5	942	182.25	119.5	0.6187	879.25	0.0006	759.75	<.0001
HT	SUR	1294	1870.75	305.5	-576.75	0.0311	988.5	<.0001	1565.25	<.0001

Table S8. TREATMENT (Laser -15 vs Laser 60 min min) stratified by ARM and TYPE

ARM	Type	T1-Laser-15 min	T2-Laser-60 min	T1-T2	*p*-value
CTRL	LAT	418	660.03	-242.03	0.0406
CTRL	MED	508.75	309.35	199.4	0.2393
CTRL	SUR	236.5	486	-249.5	0.0275
HT	LAT	1336	2142.35	-806.35	0.2631
HT	MED	716.75	1061.5	-344.75	0.2066
HT	SUR	801	1294	-493	0.0364

Table S9. TREATMENT (Thermistor-15 vs Thermistor-60 min), stratified by ARM and TYPE.

ARM	Type	T3-Thermistor-15 min	T4-Thermistor-60 min	T3-T4	*p*-value
CTRL	LAT	575	258.75	316.25	<.0001
CTRL	MED	352	269.75	82.25	0.6343
CTRL	SUR	262.75	277.75	-15	0.8694
HT	LAT	1140.5	1572.75	-432.25	0.1767
HT	MED	711.75	942	-230.25	0.3143
HT	SUR	725.83	1870.75	-1144.93	<.0001

Table S10. TREATMENT (Waterbath-15 vs Waterbath-60), stratified by ARM and TYPE.

ARM	Type	T5-Waterbath-15 min	T6-Waterbath-60 min	T5-T6	*p*-value
CTRL	LAT	411	317	94	0.3765
CTRL	MED	338.25	177	161.25	0.0928
CTRL	SUR	230	235.5	-5.5	0.9641
HT	LAT	545.75	456.75	89	0.6462
HT	MED	455	182.25	272.75	0.03
HT	SUR	409.75	305.5	104.25	0.4481

Table S11. TYPE (LAT vs MED): stratified by ARM and TREATMENT.

Treatment	ARM	LAT	MED	LAT-MED	*p*-value
T1-Laser-15 min	CTRL	418	508.75	-90.75	0.5607
T1-Laser-15 min	HT	1336	716.75	619.25	0.0028
T2-Laser-60 min	CTRL	660.03	309.35	350.68	0.0096
T2-Laser-60 min	HT	2142.35	1061.5	1080.85	0.1453
T3-Thermistor-15 min	CTRL	575	352	223	0.1986
T3-Thermistor-15 min	HT	1140.5	711.75	428.75	0.1085
T4-Thermistor-60 min	CTRL	258.75	269.75	-11	0.8513
T4-Thermistor-60 min	HT	1572.75	942	630.75	0.0289
T5-Waterbath-15 min	CTRL	411	338.25	72.75	0.4119
T5-Waterbath-15 min	HT	545.75	455	90.75	0.4238
T6-Waterbath-60 min	CTRL	317	177	140	0.2131
T6-Waterbath-60 min	HT	456.75	182.25	274.5	0.1726

Table S12. Post-hoc power analysis for *in vivo* fluorescence (SUR) for heated tumors (HT), comparing the different heating methods for 15 min and 60 min HT.

**HT Duration**	**Comparison**	**Power**
15 min	laser vs. thermistor	6.8%
15 min	laser vs. water bath	72.3%
15 min	thermistor vs. water bath	42.3%
60 min	laser vs. thermistor	57.8%
60 min	laser vs. water bath	99.2%
60 min	thermistor vs. water bath	100%

Table S13. PK by treatment (15 min)

T1-Laser-15 min	T3-Thermistor-15 min	T5-Waterbath-15 min	T1-T3	*p*-value	T1-T5	*p*-value	T3-T5	*p*-value
41.58	39.28	4.00	2.30	0.7975	37.58	<.0001	35.28	<.0001

Table S14. PK by treatment (60 min)

T2-Laser-60 min	T4-Thermistor-60 min	T6-Waterbath-60 min	T2-T4	*p*-value	T2-T6	*p*-value	T4-T6	*p*-value
17.53	21.43	1.60	-3.90	0.593	15.93	0.004	19.83	0.0002

References

[1] D. J. Schutt and D. Haemmerich, "Effects of variation in perfusion rates and of perfusion models in computational models of radio frequency tumor ablation," *Medical Physics* vol. 35, no. 8, pp. 3462-70, 2008.

[2] A. Gasselhuber *et al.*, "Targeted drug delivery by high intensity focused ultrasound mediated hyperthermia combined with temperature-sensitive liposomes: computational modelling and preliminary in vivo validation," (in eng), *Int J Hyperthermia,* vol. 28, no. 4, pp. 337-48, 2012.

[3] S. Brown, J. Hunt, and R. Hill, "Differential thermal sensitivity of tumour and normal tissue microvascular response during hyperthermia," *International journal of hyperthermia,* vol. 8, no. 4, pp. 501-514, 1992.

[4] F. P. Incropera, D. P. Dewitt, T. L. Bergman, and A. S. Lavine, *Fundamentals of Heat and Mass transfer*, Sixth ed. John Wiley and Sons, 2007, p. 917.

[5] S. T. Yang *et al.*, "Comparing the use of mid-infrared versus far-infrared lasers for mitigating damage growth on fused silica," *Applied Optics,* vol. 49, no. 2606-2616, 2010.

[6] J. R. Speakman, "Measuring energy metabolism in the mouse – theoretical, practical, and analytical considerations," *Frontiers in Physiology,* vol. 4, pp. 1-23, 2013.

[7] A. W. El-Kareh and T. W. Secomb, "Two-mechanism peak concentration model for cellular pharmacodynamics of Doxorubicin," (in eng), *Neoplasia,* vol. 7, no. 7, pp. 705-13, Jul 2005.

[8] M. R. Dreher, W. G. Liu, C. R. Michelich, M. W. Dewhirst, F. Yuan, and A. Chilkoti, "Tumor vascular permeability, accumulation, and penetration of macromolecular drug carriers," *Journal Of The National Cancer Institute,* vol. 98, no. 5, pp. 335-344, Mar 1 2006.

[9] A. W. El-Kareh and T. W. Secomb, "A mathematical model for comparison of bolus injection, continuous infusion, and liposomal delivery of doxorubicin to tumor cells," *Neoplasia,* vol. 2, no. 4, pp. 325-38, Jul-Aug 2000.

[10] A. Gasselhuber, M. R. Dreher, F. Rattay, B. J. Wood, and D. Haemmerich, "Comparison of conventional chemotherapy, stealth liposomes and temperature-sensitive liposomes in a mathematical model," (in eng), *PLoS One,* vol. 7, no. 10, p. e47453, 2012.

[11] M. W. Dewhirst and T. W. Secomb, "Transport of drugs from blood vessels to tumour tissue," *Nat Rev Cancer,* vol. 17, no. 12, pp. 738-750, Dec 2017.

[12] C. Rossmann, M. A. McCrackin, K. E. Armeson, and D. Haemmerich, "Temperature sensitive liposomes combined with thermal ablation: Effects of duration and timing of heating in mathematical models and in vivo," *PLoS ONE,* vol. 12, no. 6, p. e0179131, 2017.

[13] W. J. van der Vijgh, P. A. Maessen, and H. M. Pinedo, "Comparative metabolism and pharmacokinetics of doxorubicin and 4'-epidoxorubicin in plasma, heart and tumor of tumor-bearing mice," (in eng), *Cancer Chemother Pharmacol,* vol. 26, no. 1, pp. 9-12, 1990.

[14] A. Motamarry *et al.*, "Real-time fluorescence imaging for visualization and drug uptake prediction during drug delivery by thermosensitive liposomes," *Int J Hypertherm,* vol. 36, no. 1, pp. 816-825, 2019.

[15] P. S. Tofts *et al.*, "Estimating kinetic parameters from dynamic contrast-enhanced T(1)-weighted MRI of a diffusable tracer: standardized quantities and symbols," (in eng), *J Magn Reson Imaging,* vol. 10, no. 3, pp. 223-32, Sep 1999.

[16] D. M. Brizel, B. Klitzman, J. M. Cook, J. Edwards, G. Rosner, and M. W. Dewhirst, "A comparison of tumor and normal tissue microvascular hematocrits and red cell fluxes in a rat window chamber model," (in eng), *Int J Radiat Oncol Biol Phys,* vol. 25, no. 2, pp. 269-76, Jan 15 1993.

[17] R. T. Poon and N. Borys, "Lyso-thermosensitive liposomal doxorubicin: a novel approach to enhance efficacy of thermal ablation of liver cancer," (in eng), *Expert Opin Pharmacother,* vol. 10, no. 2, pp. 333-43, Feb 2009.

[18] A. A. Manzoor *et al.*, "Overcoming Limitations in Nanoparticle Drug Delivery: Triggered, Intravascular Release to Improve Drug Penetration into Tumors," *Cancer Res.,* vol. 72, pp. 5566-5575, 2012.

[19] R. Savai *et al.*, "Evaluation of Angiogenesis Using Micro-Computed Tomography in a Xenograft Mouse Model of Lung Cancer," *Neoplasia,* vol. 11, pp. 48-56, 2009.

[20] R. K. Jain, "Transport of molecules in the tumor interstitium: a review," (in eng), *Cancer Res,* vol. 47, no. 12, pp. 3039-51, Jun 15 1987.

[21] R. P. Brown, M. D. Delp, S. L. Lindstedt, L. R. Rhomberg, and R. P. Beliles, "Physiological parameter values for physiologically based pharmacokinetic models," *Toxicology and Industrial Health,* vol. 13, pp. 407-481, 1997.

[22] L. T. Baxter, H. Zhu, D. G. Mackensen, and R. K. Jain, "Physiologically Based Pharmacokinetic Model for Specific and Nonspecific Monoclonal Antibodies and Fragments in Normal Tissues and Human Tumor Xenografts in Nude Mice," *Cancer Research,* vol. 54, pp. 1517-1528.

[23] H. Kojima, N. Tanigawa, A. Komemushi, S. Kariya, and S. Sawada, "Computed tomography perfusion of the liver: assessment of pure portal blood flow studied with CT perfusion during superior mesenteric arterial portography," (in eng), *Acta Radiol,* vol. 45, no. 7, pp. 709-15, Nov 2004.

[24] M. Doury *et al.*, "Quantification of tumor perfusion using dynamic contrast-enhanced ultrasound: impact of mathematical modeling," *Physics in Medicine and Biology,* vol. 62, pp. 1113-1125, 2017.

[25] J. Urzova and M. Jelinek, "Heat transfer modelling of pulsed laser-tissue interaction," *Laser Phys,* vol. 28, p. 036001, 2018.

[26] P. R. Bargo *et al.*, "In vivo determination of optical properties of normal and tumor tissue with white light reflectance and an empirical light transport model during endoscopy," *Journal of Biomedical Optics* vol. 10, no. 3, p. 034018, 2005.

[27] F. Qian, N. Stowe, E. H. Liu, G. M. Saidel, and J. Gao, "Quantification of in vivo doxorubicin transport from PLGA millirods in thermoablated rat livers," *Journal of Controlled Release* vol. 2003, no. 91, pp. 157-66.

[28] D. J. Kerr, A. M. Kerr, R. I. Freshney, and S. B. Kaye, "Comparative Intracellular Uptake of Adriamycin and 4'-deoxydoxorubicin by Non-Small Cell Lung Tumor Cells in Culture and Its Relationship to Cell Survival," *Biochem Pharmacol,* vol. 35, pp. 2817-2823, 1986.

[29] A. W. El-Kareh and T. W. Secomb, "Two-mechanism peak concentration model for cellular pharmacodynamics of Doxorubicin," *Neoplasia* vol. 7, pp. 705-13, 2005.

[30] P. R. Amestoy, I. S. Duff, and J. Y. Excellent, "Multifrontal parallel distributed symmetric and unsymmetric solvers," *Computer Methods in Applied Mechanics and Engineering,* vol. 184, pp. 501-20, 2000.

[31] A. W. El-Kareh and T. W. Secomb, "A mathematical model for comparison of bolus injection, continuous infusion, and liposomal delivery of doxorubicin to tumor cells," *Neoplasia* vol. 2, pp. 325-338, 2000.

[32] A. L. Bredlau *et al.*, "Localized delivery of therapeutic doxorubicin dose across the canine blood-brain barrier with hyperthermia and temperature sensitive liposomes," *Drug Deliv,* vol. 25, no. 1, pp. 973-984, Nov 2018.

[33] A. Motamarry *et al.*, "Real-time fluorescence imaging for visualization and drug uptake prediction during drug delivery by thermosensitive liposomes," *International Journal of Hyperthermia,* vol. 36, no. 1, pp. 817-826, 2019/01/01 2019.
